# Clinical, Endoscopic, and Pathologic Spectrum of Pediatric Polyps: A Single-Center Study in the Current Polypectomy Era

**DOI:** 10.3390/jcm15031061

**Published:** 2026-01-29

**Authors:** Sevim Çakar, Betül Aksoy, Oğuzhan Akyaz, Tuğçe Tatar Arık, Süleyman Dolu, Mesut Akarsu, Safiye Aktaş, Yeşim Öztürk

**Affiliations:** 1 Department of Pediatrics, Division of Pediatric Gastroenterology, Faculty of Medicine, Dokuz Eylül University, Izmir 35340, Turkey; 2 Department of Internal Medicine, Division of Gastroenterology, Faculty of Medicine, Dokuz Eylül University, Izmir 35340, Turkey; 3 Department of Pathology, Faculty of Medicine, Dokuz Eylül University, Izmir 35340, Turkey

**Keywords:** colorectal polyps, pediatric endoscopy, polyposis syndromes, sessile polyps, adenomatous polyps, juvenile polyps

## Abstract

**Background:** Pediatric gastrointestinal polyps represent a heterogeneous entity with variable clinical behavior, ranging from solitary benign lesions to syndromic forms associated with significant malignant potential. This study provides contemporary data, including upper GI and small-bowel polyps, with an unusually high syndromic yield (27.6%) compared to prior pediatric cohorts. **Methods:** This retrospective single-center study included children aged 0–18 years who underwent esophagogastroduodenoscopy and/or colonoscopy and were diagnosed with at least one gastrointestinal polyp between January 2015 and October 2025. Demographic characteristics, presenting symptoms, endoscopic features, histopathology, management strategies, and status of polyposis syndrome were collected. Statistical analyses were performed using IBM SPSS Statistics 27.0, with a significance threshold of *p* < 0.05. **Results:** Seventy-six patients (mean age 10.6 ± 5.0 years; 47.4% female) were evaluated. Gastrointestinal bleeding was the most common presenting symptom (37.1%). Solitary (63.2%) and sessile (59.2%) polyps predominated, with a median size of 7.0 mm (IQR 3.2–20.0). Juvenile (28.9%) and inflammatory (22.4%) polyps were the most frequent histologic subtypes. Polyposis syndromes were identified in 27.6% of patients and were significantly associated with multiple polyps (*p* < 0.001), proximal or intestinal distribution (*p* < 0.001), and adenomatous or hamartomatous histology (*p* < 0.001). Endoscopic polypectomy was successful in 94.7% of cases, with no major complications reported. **Conclusions:** Given the 27.6% prevalence of polyposis syndromes observed in this cohort, pediatric gastrointestinal polyps cannot be assumed to be uniformly benign. Our findings support comprehensive endoscopic evaluation, routine histopathology, and early genetic referral, specifically in patients with multiple, proximal, or mixed-morphology polyps.

## 1. Introduction

Pediatric gastrointestinal polyps are among the most frequent organic causes of lower gastrointestinal bleeding in children, yet they represent a surprisingly heterogeneous group in terms of histology, distribution, and malignant potential. Although the classic solitary juvenile polyp in a preschool child has long been regarded as a benign, self-limited lesion, contemporary series underscore that a substantial subset of pediatric patients harbor multiple, proximally located, or syndromic polyps that carry significant long-term cancer risk [1,2,3].

Population-based and single-center studies from North America, Europe, and Asia consistently report juvenile polyps as the dominant histologic subtype and rectal bleeding as the most common presentation, but with substantial variability in age at diagnosis, sex distribution, multiplicity, and colonic location [4,5,6,7,8]. Emerging work has also highlighted potentially modifiable factors and familial aggregation as correlates of pediatric colorectal polyps, while regional series suggest that endoscopy access and referral pathways may influence case mix and therapeutic practice patterns [9,10].

In parallel, the diagnostic and management landscape has rapidly evolved. ESPGHAN position papers and complementary US Multi-Society Task Force guidance provide structured recommendations for genetic evaluation and risk-adapted surveillance across major pediatric polyposis syndromes, reinforcing the role of early recognition in initiating lifelong cancer-prevention strategies [11,12,13,14]. At the procedural level, contemporary pediatric endoscopy increasingly favors cold snare polypectomy for diminutive and small lesions, reserving hot snare techniques and endoscopic mucosal resection (EMR) for larger or complex polyps; quality initiatives further support the overall safety of therapeutic endoscopy in children, while acknowledging persistent—albeit low—procedure-related risks in medically complex patients [15,16,17,18,19].

Despite this expanding evidence base, important gaps remain. Many foundational pediatric polyp series predate contemporary guideline frameworks and the widespread adoption of modern polypectomy techniques and pediatric endoscopy quality indicators [1,4,20,21]. Single-center data from varied geographic and socioeconomic contexts therefore remain essential to characterize real-world patterns of presentation, polyp distribution, histology, and syndromic yield [7,8,9].

In this context, we conducted a single-center study of pediatric gastrointestinal polyps to comprehensively describe the clinical presentation, endoscopic and histologic characteristics, and management of affected children and adolescents in the era of modern pediatric endoscopy and polyposis guidelines.

## 2. Materials and Methods

### 2.1. Study Design and Setting

This retrospective descriptive study was conducted at the Department of Pediatric Gastroenterology, Hepatology, and Nutrition, Dokuz Eylul University Faculty of Medicine, a tertiary academic center in Türkiye. The study evaluated pediatric patients who underwent endoscopic procedures between January 2015 and October 2025.

We used the cold or hot snare polypectomy for small lesions and hot snare for larger or sessile polyps, performed by experienced pediatric gastroenterologists. The histopathological evaluation was performed by experienced gastrointestinal pathologists, with review independent of clinical and endoscopic findings.

### 2.2. Study Population

Children aged 0–18 years who underwent esophagogastroduodenoscopy and/or colonoscopy and were diagnosed with at least one gastrointestinal or colorectal polyp were eligible for inclusion. Patients with incomplete clinical, endoscopic, radiological, or histopathological data were excluded.

A priori power analysis using G*Power 3.1 determined a minimum sample size of 52 (effect size w = 0.50, α = 0.05, power = 80%).

### 2.3. Data Collection

Data were retrieved from electronic medical records, endoscopy reports, histopathology archives, and imaging files. Variables collected included demographic features, clinical presentation, endoscopic and laboratory findings, histopathology, management details, genetic evaluation, and follow-up data, including recurrence.

Genetic testing was performed selectively in patients with clinical suspicion of polyposis syndromes (multiple polyps, proximal/small bowel involvement, family history). Polyposis syndromes were defined based on clinical, endoscopic, and histopathological criteria when genetic confirmation was unavailable.

### 2.4. Outcome Measures

Primary outcomes included clinical, endoscopic, and histopathologic characteristics of polyps.

### 2.5. Statistical Analysis

Data was analyzed using IBM SPSS Statistics version 27.0. Categorical variables were expressed as frequencies and percentages. Continuous variables were summarized as mean ± SD, median, minimum, and maximum. Normality was assessed using the Shapiro–Wilk test and Q–Q plots. Group comparisons used *t*-tests or Mann–Whitney U tests as appropriate. Associations between categorical variables were evaluated with chi-square tests. A *p*-value < 0.05 was considered statistically significant.

## 3. Results

A total of 76 pediatric patients with gastrointestinal polyps were included. The mean age was 10.6 ± 5.0 years, and 47.4% were female. The most commonly present complaint was gastrointestinal bleeding (37.1%), followed by abdominal pain (12.9%) and functional dyspepsia (11.4%) (Table 1). A positive family history was documented in 28.0% of patients. Polyposis syndromes were identified in 27.6% of the cohort, most commonly Peutz–Jeghers syndrome (17.1%) and familial adenomatous polyposis (7.9%) (Table 1).

Sessile morphology was the predominant polyp type (59.2%), and 63.2% of patients presented with a single lesion (Table 2). The median polyp size was 7.0 mm (IQR 3.2–20.0). The left colon was the most frequent location (43.4%), followed by gastric (19.7%) and intestinal (18.5%) sites (Table 2). Management was primarily endoscopic (94.7%), with only a minority requiring combined or surgical intervention. Double-balloon enteroscopy was performed in 9 patients, of whom only one was non-syndromic (gastrointestinal bleeding) (Table 2).

Histopathological evaluation demonstrated a predominance of juvenile (28.9%) and inflammatory (22.4%) polyps, followed by hyperplastic (17.1%) and adenomatous (15.8%) pathology (Table 3). Additional pathological findings were observed in 63.2% of upper endoscopies and 68.4% of colonoscopies (Table 3).

The size of polyps did not differ significantly between patients with and without polyposis syndromes (median 6.0 mm [IQR 3–30] vs. 7.0 mm [IQR 4–12], *p* = 0.486). There was a significant association between polyposis syndrome status and polyp morphology (χ^2^ = 9.12, *p* = 0.010), with mixed sessile–pedunculated polyps occurring more frequently in syndromic patients, whereas sessile lesions predominated in non-syndromic cases (Table 4). There was a highly significant association between polyposis syndrome status and histopathological subtype (χ^2^ = 58.7, *p* < 0.001), with adenomatous and hamartomatous lesions predominating in syndromic patients, whereas juvenile and inflammatory polyps were almost exclusively observed in non-syndromic cases (Table 4). There was a highly significant association between polyp location and polyposis syndrome status (χ^2^ = 27.2, *p* < 0.001), with intestinal involvement predominating in syndromic patients and left-sided lesions occurring mainly in non-syndromic cases (Table 4).

The prevalence of anemia did not differ significantly between syndromic and non-syndromic patients (anemia present: 13 vs. 37; anemia absent: 8 vs. 17; *p* = 0.785). Likewise, MCV values were similar between groups, with a median of 80.0 fL (IQR 72.0–84.3) in syndromic patients and 77.7 fL (IQR 73.1–81.0) in non-syndromic patients (*p* = 0.409).

Polyp size varied across anatomical locations, with the largest median size observed in intestinal polyps (12.5 mm, IQR 5.0–37.5) and the smallest in pan-colonic involvement (3.0 mm, IQR 3.0–5.25). This difference approached statistical significance (Kruskal–Wallis *p* = 0.050). While polyp size differed across locations at a global level (Kruskal–Wallis *p* = 0.050), post hoc pairwise testing with Bonferroni correction revealed no statistically significant differences between any specific location groups (all adjusted *p* > 0.05).

Atypical but clinically relevant cases included hyperplastic polyps associated with anal verruca (10 mm) and sclerosing cholangitis (multiple 3 mm), a radiology-prompted colonoscopy showing a 5 cm tubulovillous sigmoid polyp with subsequent T-cell lymphoma, and an incidental 3 cm hyperplastic gastric polyp detected during evaluation for portal hypertension. A 14-year-old male from a Peutz–Jeghers syndrome family presented with intussusception and was diagnosed with colorectal adenocarcinoma. In inflammatory bowel disease (n = 11), one patient had a hyperplastic gastric polyp and ten had colonic inflammatory polyps (3–10 mm).

No major complications related to polypectomy were observed during or after the procedures.

## 4. Discussion

This single-center cohort provides contemporary data on the clinical and histopathological profile of pediatric gastrointestinal polyps, demonstrating a pattern largely characterized by solitary, distally located, and benign lesions. The predominance of solitary polyps and left-sided distribution aligns with recent pediatric cohorts in which rectosigmoid juvenile polyps represent the most frequent phenotype [8,9]. In accordance with these reports, hematochezia emerged as the principal presenting symptom in our population, underscoring its continued value as a key clinical marker of colorectal polyps in childhood [8,9]. Importantly, however, our data also highlight a clinically distinct subgroup that deviates from the “classic” profile—patients presenting with multiplicity, proximal disease, and syndromic background—illustrating that a distal-only diagnostic framework cannot fully capture the contemporary pediatric polyp spectrum.

Juvenile polyps represent a specific subtype of hamartomatous polyps; thus, while most solitary juvenile polyps are sporadic and benign, multiple hamartomatous polyps should raise suspicion for an underlying hamartomatous polyposis syndrome. The histologic predominance of juvenile and inflammatory polyps is consistent with prior pediatric studies reporting a largely benign pathology profile [6,7].

Nevertheless, the identification of adenomatous components in a subset of patients is clinically meaningful, as current consensus emphasizes that both adenomatous lesions and hamartomatous polyposis syndromes carry elevated long-term malignancy risk and therefore require structured surveillance strategies [14]. This position is reinforced by expert guidance highlighting that syndromic entities such as juvenile polyposis syndrome, familial adenomatous polyposis, and Peutz–Jeghers syndrome often demonstrate extensive polyp burden, proximal distribution, and higher-risk histologic patterns compared with sporadic disease [11,12,13]. Within this context, the finding that our syndromic yield exceeded rates reported in several contemporary pediatric cohorts [9,22] warrants interpretation beyond simple description.

A key observation of this study is the unexpectedly high prevalence of polyposis syndromes, suggesting that syndromic disease may be more common in “real-world” tertiary pediatric endoscopy populations than historically assumed. Several mechanisms may plausibly explain this enrichment. First, referral bias is inherent to tertiary centers, where children with red-flag features—including early onset symptoms, family history, anemia, multiple lesions, or atypical distribution—are more likely to be evaluated and to undergo comprehensive endoscopic work-up and genetic assessment [11,12,13]. Second, increasing clinician awareness and lowering thresholds for genetic evaluation may enhance detection of syndromic cases that might previously have been classified as sporadic, particularly when the phenotype is subtle (e.g., limited polyp number at presentation, or mixed histology) [11,12,13]. Third, our cohort included clinically meaningful outliers—such as proximal and small-bowel involvement and malignancy-associated presentations—which may represent the “tip of the iceberg” of syndromic risk within pediatric polyp populations. Collectively, these observations emphasize that syndromic disease is not merely a rare exception; rather, it constitutes a high-impact subgroup whose identification can substantially alter long-term management, surveillance intensity, and family-based risk assessment [11,12,13,14].

The anatomic distribution of polyps in our cohort further strengthens this interpretation. While distal lesions predominated overall, syndromic cases displayed a stronger association with multiplicity and proximal or intestinal involvement, consistent with guidance that proximal disease is frequently under-recognized when limited examinations are performed [11,12,13]. This has direct practical implications: reliance on sigmoidoscopy or incomplete colonoscopy risks underestimating polyp burden, missing proximal lesions with distinct histology, and delaying recognition of an underlying polyposis syndrome. Our findings therefore support the increasing consensus that pancolonoscopy should be favored over limited sigmoidoscopy in children with suspected or high-risk presentations, including those with multiple polyps, anemia, recurrent bleeding, family history, or atypical endoscopic appearance [9,18]. Importantly, this approach is not simply diagnostic; it is also prognostic, because accurate mapping of lesion distribution is essential for defining surveillance intervals, determining the need for genetic evaluation, and planning long-term multidisciplinary follow-up [11,12,13,14].

Beyond the colon, the presence of upper gastrointestinal and small-bowel involvement represents one of the most clinically consequential signals in this cohort. Small-bowel polyps are particularly relevant in syndromes such as Peutz–Jeghers, where intussusception and malignancy risks are central complications, and where clinically significant lesions may be missed without targeted evaluation [11,12,13,14,18]. Our cohort’s inclusion of patients with proximal and small-bowel disease underscores that pediatric polyp care increasingly extends beyond colonoscopy into a broader, syndrome-informed approach to gastrointestinal surveillance. Clinically, these findings suggest that “negative colonoscopy” should not always be interpreted as reassuring when the pre-test probability of syndromic disease is high. Instead, symptom burden, family history, and phenotype should guide further investigation of the upper GI tract and small bowel, particularly in patients with multiple lesions or suggestive histopathology [11,12,13,18].

In this evolving landscape, enteroscopy—including advanced modalities such as double-balloon enteroscopy—may have an expanding role in pediatric practice. While most sporadic polyps are effectively managed by standard colonoscopic resection, syndromic patients often accumulate lesions across multiple segments over time, leading to repeated interventions, bleeding, anemia, and obstructive complications [14,18]. Enteroscopy provides an opportunity for both diagnostic mapping and therapeutic polypectomy within the small bowel, potentially reducing the morbidity of recurrent intussusception and minimizing the need for repeated surgical resections in selected high-risk cases [14,18]. Our data reinforce the practical implication that tertiary centers managing pediatric polyposis should consider structured pathways that include access to advanced endoscopic expertise when phenotype and distribution suggest small-bowel involvement. These findings also argue for a surveillance paradigm that is individualized, risk-stratified, and multidisciplinary—linking pediatric gastroenterology, genetics, pathology, and when needed, surgical teams—to support early detection and complication prevention across childhood and adolescence [11,12,13,14].

Therapeutically, the high success rate of endoscopic polypectomy in this study aligns with evidence that pediatric endoscopic resection can be performed safely with low complication rates in expert settings [16,17]. However, our cohort also illustrates a critical distinction: the technical success of single-polyp resection does not equate to completion of care in syndromic disease. For polyposis syndromes, endoscopic treatment must be integrated into long-term surveillance planning, recognition of cumulative risk, and repeated evaluation of disease extent over time [14,18]. In other words, a syndromic diagnosis fundamentally shifts the clinical endpoint from “polyp removal” to “lifelong risk management,” with implications for both patients and families [11,12,13,14].

Taken together, our findings reinforce contemporary understanding that while most pediatric colorectal polyps are benign and distal, a clinically distinct subgroup characterized by syndromic background, multiplicity, proximal distribution, and higher-risk histology requires early recognition, genetic assessment, and longitudinal multidisciplinary management [11,13,14]. This subgroup is precisely where comprehensive endoscopic assessment—including pancolonoscopy and selective evaluation of the upper GI tract and small bowel—may deliver the greatest clinical value, preventing missed diagnoses and enabling risk-tailored surveillance [9,18]. Future prospective multicenter studies incorporating standardized genetic testing and harmonized surveillance protocols are needed to refine risk stratification and to determine which clinical thresholds should prompt advanced small-bowel evaluation and enteroscopy in pediatric polyposis populations.

The limitations of this study, including its cross-sectional retrospective design and relatively small sample size, must be acknowledged. Additional limitations include referral bias inherent to a tertiary center, selective genetic testing potentially underestimating mutation rates, retrospective heterogeneity in polypectomy techniques and operators, and lack of a standardized surveillance protocol. Nevertheless, despite these constraints, the study provides clinically relevant contemporary insights—particularly regarding syndromic yield and the clinical importance of proximal and small-bowel disease—that support a more comprehensive and syndrome-aware approach to pediatric polyp evaluation and follow-up.

## 5. Conclusions

In agreement with recent literature, this study confirms that pediatric gastrointestinal polyps are predominantly solitary, left-sided, and histologically benign [8,9,22]. However, the strong association between polyposis syndromes and multiple, proximally located, and adenomatous or hamartomatous lesions underscores the need for risk stratification and vigilant lifelong surveillance [11,13,14].

Accordingly:

1. **Pancolonoscopy** should be considered the preferred initial diagnostic approach in symptomatic or high-risk patients.
2. **Histopathologic evaluation** remains essential for determining malignant potential.
3. **Syndromic cases** require genetic counseling and structured long-term follow-up.
4. **Endoscopic polypectomy** is a safe and effective first-line treatment in appropriately selected patients.

Overall, these findings highlight the heterogeneous nature of pediatric polyps and emphasize the importance of early recognition, comprehensive endoscopic evaluation, and multidisciplinary management in children at risk for polyposis syndromes.

## Figures and Tables

**Table 1:** Demographic and Clinical Characteristics of Children with Polyps.

Variable	Number (%)
Age, years	10.6 ± 5.0
Sex	Female: 36 (47.4%)
	Male: 40 (52.6%)
Presenting complaint *	Gastrointestinal bleeding: 26 (37.1%)
	Abdominal pain: 9 (12.9%)
	Functional dyspepsia: 8 (11.4%)
	Rectal prolapse: 5 (7.1%)
	Chronic iron deficiency anemia: 5 (7.1%)
	Weight loss: 4 (5.7%)
	Invagination: 4 (5.7%)
	Diarrhea: 3 (4.3%)
	Constipation: 1 (1.4%)
	Incidental: 2 (2.9%)
	Family screening: 2 (2.9%)
	Detection of mass in imaging: 1 (1.4%)
Family history *	Positive: 21 (28.0%)
	Negative: 54 (72.0%)
Diagnosis of polyposis syndrome	Peutz–Jeghers syndrome: 13 (17.1%)
	Familial adenomatous polyposis: 6 (7.9%)
	Turcot syndrome: 1 (1.3%)
	Gardner syndrome: 1 (1.3%)
	No defined syndrome: 55 (72.4%)
Detected mutation of polyposis syndrome	6 (7.9%)
	No/Not tested: 70 (92.1%)

* Missing data present.

**Table 2:** Characteristics of Polyps at Endoscopy and Treatment.

Variable	Number (%)
Polyp type *	Sessile: 45 (59.2%)
	Pedunculated: 23 (30.3%)
	Sessile + Pedunculated: 7 (9.2%)
Number of polyps	Single: 48 (63.2%)
	Multiple: 28 (36.8%)
Polyp size, median (mm)	7.0 (IQR 3.2–20.0)
Polyp location	Left colon: 32 (42.1%)
	Pan-colonic: 8 (10.5%)
	Right colon: 6 (7.9%)
	Gastric: 15 (19.7%)
	Intestinal: 14 (18.4%)
Number of polypectomies	Median 1.0 (IQR 1.0–1.5)
Polypectomy technique	Endoscopic: 72 (94.7%)
	Surgical + Endoscopic: 3 (3.9%)
	Surgical: 1 (1.4%)
Double-balloon enteroscopy	Performed: 9 (11.8%)
	Not performed: 67 (88.2%)

* Missing data present.

**Table 3:** Histopathology and Endoscopic Findings of Polyps.

Variable	Number (%)
Histopathology findings	Adenomatous: 12 (15.8%)
	Hamartomatous: 10 (13.2%)
	Juvenile: 22 (28.9%)
	Adenomatous + Hamartomatous: 1 (1.3%)
	Inflammatory: 17 (22.4%)
	Hyperplastic: 13 (17.1%)
	Tubulovillous: 1 (1.3%)
Additional pathological upper endoscopic findings	Present: 48 (63.2%)
	Absent: 28 (36.8%)
Additional pathological colonoscopic findings	Present: 52 (68.4%)
	Absent: 24 (31.6%)

**Table 4:** Polyp Location, Morphology, and Histopathology by Polyposis Syndrome Status.

Variable		No Syndrome (n = 55)	Polyposis Syndrome (n = 21)	*p*-Value
Polyp Morphology	Sessile	32	13	0.010
	Pedunculated	20	3	
	Mixed (Sessile + Pedunculated)	2	5	
Histopathology	Adenomatous	2	9	<0.001
	Hamartomatous	1	9	
	Juvenile	22	0	
	Inflammatory	17	0	
	Hyperplastic	12	1	
Polyp Location	Left colon	29	3	<0.001
	Pan-colon	4	4	
	Right colon	6	0	
	Stomach	13	2	
	Intestinal	2	12	
Number of Polyps, Median (IQR)		1 (1–1)	6 (2–6)	<0.001

## Data Availability

The corresponding author will share the data upon request due to legal and ethical reasons.
